# *N*-Benzyl-2-hydroxy­benzamide

**DOI:** 10.1107/S1600536808010453

**Published:** 2008-04-23

**Authors:** Qiu-Xia Zhang, Bi-Song Zhang

**Affiliations:** a College of Materials Science and Chemical Engineering, Jinhua College of Profession and Technology, Jinhua, Zhejiang 321017, People’s Republic of China

## Abstract

In the title compound, C_14_H_13_NO_2_, the mean planes through the benzyl and 2-hydoxybenzamide units make a dihedral angle of 68.81 (7)°. There is an intra­molecular O—H⋯O hydrogen bond involving the carbonyl O atom and the 2-hydr­oxy substituent. In the crystal structure, N—H⋯O hydrogen bonds link symmetry-related mol­ecules into one-dimensional chains extending along the *a*-axis direction. These chains are further connected *via* C—H⋯O hydrogen bonds, forming a sheet-like structure

## Related literature

For related literature, see: Agwade (1982 ▶); Allen *et al.* (1987 ▶).
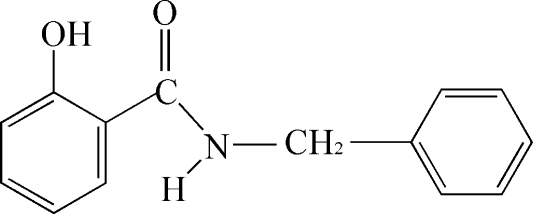


## Experimental

### 

#### Crystal data

C_14_H_13_NO_2_
*M* *_r_* = 227.25
Monoclinic, 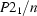

*a* = 12.478 (3) Å
*b* = 8.3503 (17) Å
*c* = 12.664 (3) Å
β = 118.02 (3)°
*V* = 1164.9 (6) Å^3^
*Z* = 4
Mo *K*α radiation
μ = 0.09 mm^−1^
*T* = 290 (2) K
0.33 × 0.22 × 0.20 mm

#### Data collection

Rigaku R-AXIS RAPID diffractometer
Absorption correction: multi-scan (*ABSCOR*; Higashi, 1995 ▶) *T*_min_ = 0.977, *T*_max_ = 0.983
11112 measured reflections
2665 independent reflections
1648 reflections with *I* > 2σ(*I*)
*R*_int_ = 0.037

#### Refinement

*R*[*F* ^2^ > 2σ(*F* ^2^)] = 0.043
*wR*(*F* ^2^) = 0.116
*S* = 1.03
2665 reflections
207 parameters
All H-atom parameters refined
Δρ_max_ = 0.17 e Å^−3^
Δρ_min_ = −0.14 e Å^−3^

### 

Data collection: *RAPID-AUTO* (Rigaku, 1998 ▶); cell refinement: *RAPID-AUTO*; data reduction: *CrystalStructure* (Rigaku/MSC, 2002 ▶); program(s) used to solve structure: *SHELXS97* (Sheldrick, 2008 ▶); program(s) used to refine structure: *SHELXL97* (Sheldrick, 2008 ▶); molecular graphics: *SHELXTL* (Sheldrick, 2008 ▶); software used to prepare material for publication: *SHELXL97*.

## Supplementary Material

Crystal structure: contains datablocks I, global. DOI: 10.1107/S1600536808010453/su2048sup1.cif

Structure factors: contains datablocks I. DOI: 10.1107/S1600536808010453/su2048Isup2.hkl

Additional supplementary materials:  crystallographic information; 3D view; checkCIF report

## Figures and Tables

**Table 1:** Hydrogen-bond geometry (Å, °)

*D*—H⋯*A*	*D*—H	H⋯*A*	*D*⋯*A*	*D*—H⋯*A*
N1—H*N*1⋯O1^i^	0.882 (18)	2.083 (18)	2.9191 (18)	158.1 (18)
O1—H*O*1⋯O2	0.98 (3)	1.56 (3)	2.4886 (19)	157 (2)
C2—H2⋯O1^i^	0.956 (19)	2.58 (2)	3.507 (2)	162.5 (15)

Symmetry code: (i) 
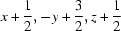
.

**Fig. 1.**
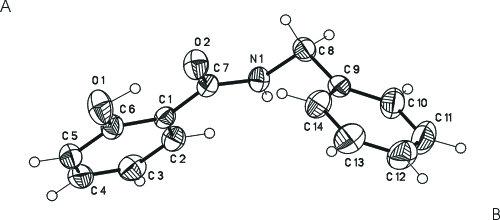
A view of the moleculecular structure of I, showing the atom-labelling scheme. Displacement ellipsoids are drawn at the 40% probability level.

**Fig. 2.**
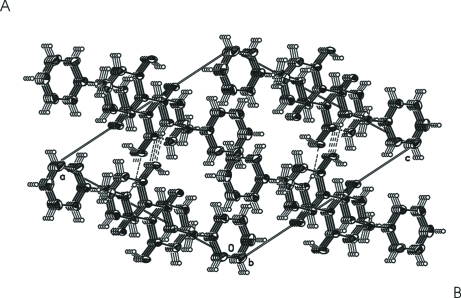
The crystal packing diagram for compound I, viewed down the b axis. Dashed lines indicate N-H···O hydrogen bonds.

## supplementary crystallographic information

### Comment

Over a quater of a century ago (Agwade, 1982) published a thesis on Potential Central Nervous System Active Agents, which included the synthesis of aromatic *N*-benzyl amides. One of the compounds synthesized was N-benzyl-2-hydroxy-benzamide, (I), whose crystal structure has not been descibed until now.

The molecular structure of compound I is illustrated in Fig. 1. The bond lengths and angles are close to normal values (Allen *et al.*, 1987). There is an intramolecular O-H···O hydrogen bond in the molecule involving the carbonyl O-atom and the 2-hydroxyl substituent (Table 1). The best planes through the benzyl (atoms C8,C9-C14) and the 2-hydoxybenzamide (atoms C1-C6,C7,N1,O1,O2) moieties are inclined to one another by 68.81 (7)°.

In the crystal structure of I symmetry related molecules are connected via an N-H···O hydrogen bond to form chains running along the a direction. These chains are further connected connected via C-H···O hydrogen bonds (Table 1) to form a sheet-like structure (Fig 2).

### Experimental

Freshly prepared CuCO_3_ (0.310 g 2.50 mmol), [C_6_H_4_(COOC_6_H_4_CONHCH_2_ph)_2_](0.350 g 0.601 mmol), 2-chloro-benzoic acid (0.160 g 1.022 mmol), and 15 ml CH_3_OH/H_2_O (1:2,*v*/*v*) were mixed and stirred for *ca*. 1.5 h. The resulting suspension was heated in a 23 ml Teflon-lined stainless steel autoclave at 373 K for 7 days. After the autoclave was cooled to room temperature, and colorless block-like crystals, suitable for X-ray analysis, were obtained.

### Refinement

All of the H atoms were located in difference Fourier syntheses and were freely refined: O-H = 0.98 (3), N-H = 0.882 (18), and C-H = 0.94 (2) - 1.010 (16) Å.

### Crystal data

**Table:**

C_14_H_13_NO_2_	*F*_000_ = 480
*M**_r_* = 227.25	*D*_x_ = 1.296 Mg m^−^^3^
Monoclinic, *P*2_1_/*n*	Mo *K*α radiation λ = 0.71073 Å
Hall symbol: -P 2yn	Cell parameters from 6913 reflections
*a* = 12.478 (3) Å	θ = 3.0–27.5º
*b* = 8.3503 (17) Å	µ = 0.09 mm^−^^1^
*c* = 12.664 (3) Å	*T* = 290 (2) K
β = 118.02 (3)º	Block, colorless
*V* = 1164.9 (6) Å^3^	0.33 × 0.22 × 0.20 mm
*Z* = 4	

### Data collection

**Table:**

Rigaku R-AXIS RAPID diffractometer	2665 independent reflections
Radiation source: fine-focus sealed tube	1648 reflections with *I* > 2σ(*I*)
Monochromator: graphite	*R*_int_ = 0.037
Detector resolution: 10 pixels mm^-1^	θ_max_ = 27.5º
*T* = 290(2) K	θ_min_ = 3.0º
ω scans	*h* = −15→16
Absorption correction: multi-scan(ABSCOR; Higashi, 1995)	*k* = −10→10
*T*_min_ = 0.977, *T*_max_ = 0.983	*l* = −16→16
11112 measured reflections	

### Refinement

**Table:**

Refinement on *F*^2^	Hydrogen site location: inferred from neighbouring sites
Least-squares matrix: full	All H-atom parameters refined
*R*[*F*^2^ > 2σ(*F*^2^)] = 0.043	*w* = 1/[σ^2^(*F*_o_^2^) + (0.0564*P*)^2^ + 0.0491*P*] where *P* = (*F*_o_^2^ + 2*F*_c_^2^)/3
*wR*(*F*^2^) = 0.116	(Δ/σ)_max_ < 0.001
*S* = 1.03	Δρ_max_ = 0.17 e Å^−^^3^
2665 reflections	Δρ_min_ = −0.14 e Å^−^^3^
207 parameters	Extinction correction: SHELXL97 (Sheldrick, 2008), Fc^*^=kFc[1+0.001xFc^2^λ^3^/sin(2θ)]^-1/4^
Primary atom site location: structure-invariant direct methods	Extinction coefficient: 0.011 (2)
Secondary atom site location: difference Fourier map	

### Special details

**Table:**

Geometry. Bond distances, angles etc. have been calculated using the rounded fractional coordinates. All su's are estimated from the variances of the (full) variance-covariance matrix. The cell esds are taken into account in the estimation of distances, angles and torsion angles
Refinement. Refinement of *F*^2^ against ALL reflections. The weighted *R*-factor *wR* and goodness of fit *S* are based on *F*^2^, conventional *R*-factors *R* are based on *F*, with *F* set to zero for negative *F*^2^. The threshold expression of *F*^2^ > σ(*F*^2^) is used only for calculating *R*-factors(gt) *etc*. and is not relevant to the choice of reflections for refinement. *R*-factors based on *F*^2^ are statistically about twice as large as those based on *F*, and *R*- factors based on ALL data will be even larger.

### Fractional atomic coordinates and isotropic or equivalent isotropic displacement parameters (Å^2^)

**Table:**

	*x*	*y*	*z*	*U*_iso_*/*U*_eq_	
O1	0.29704 (10)	0.74227 (17)	−0.23736 (9)	0.0731 (4)	
O2	0.49480 (9)	0.60089 (14)	−0.16419 (9)	0.0600 (4)	
N1	0.64585 (9)	0.63326 (15)	0.02206 (11)	0.0449 (4)	
C1	0.45255 (11)	0.76116 (16)	−0.03318 (11)	0.0396 (4)	
C2	0.48553 (14)	0.82179 (19)	0.07995 (14)	0.0493 (5)	
C3	0.40715 (15)	0.9140 (2)	0.10230 (16)	0.0592 (6)	
C4	0.29300 (15)	0.9477 (2)	0.01061 (17)	0.0604 (6)	
C5	0.25762 (14)	0.8909 (2)	−0.10172 (16)	0.0576 (6)	
C6	0.33637 (12)	0.79771 (18)	−0.12499 (13)	0.0478 (5)	
C7	0.53270 (11)	0.66022 (17)	−0.06264 (12)	0.0412 (4)	
C8	0.73259 (14)	0.5375 (2)	0.00128 (16)	0.0508 (5)	
C9	0.80422 (12)	0.63678 (17)	−0.04347 (14)	0.0460 (5)	
C10	0.92320 (15)	0.6768 (3)	0.03099 (19)	0.0722 (7)	
C11	0.98716 (18)	0.7699 (3)	−0.0108 (2)	0.0875 (9)	
C12	0.9333 (2)	0.8228 (3)	−0.1254 (2)	0.0785 (9)	
C13	0.81540 (19)	0.7850 (3)	−0.20000 (19)	0.0721 (8)	
C14	0.75139 (16)	0.6917 (2)	−0.15893 (16)	0.0596 (6)	
HN1	0.6728 (14)	0.6819 (19)	0.0917 (16)	0.059 (5)*	
H2	0.5651 (15)	0.7995 (18)	0.1431 (15)	0.060 (5)*	
HO1	0.367 (2)	0.681 (3)	−0.230 (2)	0.106 (7)*	
H3	0.4331 (15)	0.952 (2)	0.1817 (17)	0.078 (5)*	
H4	0.2386 (15)	1.009 (2)	0.0252 (15)	0.071 (5)*	
H5	0.1770 (17)	0.913 (2)	−0.1697 (16)	0.081 (5)*	
H8A	0.6833 (14)	0.455 (2)	−0.0610 (14)	0.062 (5)*	
H8B	0.7896 (14)	0.4913 (19)	0.0805 (15)	0.062 (5)*	
H10	0.9607 (17)	0.633 (2)	0.1088 (19)	0.087 (6)*	
H11	1.070 (2)	0.793 (3)	0.042 (2)	0.107 (7)*	
H12	0.9743 (19)	0.887 (3)	−0.1597 (18)	0.099 (7)*	
H13	0.7716 (18)	0.825 (3)	−0.283 (2)	0.098 (7)*	
H14	0.6696 (16)	0.668 (2)	−0.2122 (15)	0.070 (5)*	

### Atomic displacement parameters (Å^2^)

**Table:**

	*U*^11^	*U*^22^	*U*^33^	*U*^12^	*U*^13^	*U*^23^
O1	0.0512 (6)	0.1059 (10)	0.0395 (6)	0.0150 (7)	0.0024 (5)	−0.0056 (6)
O2	0.0503 (6)	0.0798 (8)	0.0421 (6)	0.0005 (5)	0.0152 (5)	−0.0147 (5)
N1	0.0376 (6)	0.0549 (8)	0.0399 (7)	0.0001 (5)	0.0162 (5)	0.0025 (6)
C1	0.0369 (6)	0.0420 (8)	0.0378 (7)	−0.0052 (6)	0.0159 (5)	0.0030 (6)
C2	0.0465 (8)	0.0557 (10)	0.0433 (8)	−0.0040 (7)	0.0190 (7)	−0.0014 (7)
C3	0.0664 (10)	0.0594 (10)	0.0604 (11)	−0.0070 (8)	0.0370 (9)	−0.0106 (8)
C4	0.0586 (10)	0.0493 (10)	0.0853 (13)	0.0013 (8)	0.0438 (9)	0.0000 (9)
C5	0.0440 (8)	0.0553 (10)	0.0696 (11)	0.0052 (7)	0.0235 (8)	0.0109 (8)
C6	0.0408 (7)	0.0533 (9)	0.0436 (8)	−0.0018 (7)	0.0150 (6)	0.0048 (7)
C7	0.0379 (7)	0.0458 (8)	0.0374 (8)	−0.0052 (6)	0.0156 (5)	0.0021 (6)
C8	0.0454 (8)	0.0501 (9)	0.0583 (10)	0.0065 (7)	0.0256 (7)	0.0099 (8)
C9	0.0447 (7)	0.0410 (8)	0.0558 (9)	0.0018 (6)	0.0266 (6)	−0.0020 (7)
C10	0.0477 (9)	0.0840 (14)	0.0753 (13)	−0.0025 (9)	0.0209 (8)	0.0173 (11)
C11	0.0510 (11)	0.0993 (17)	0.1074 (18)	−0.0151 (11)	0.0333 (11)	0.0117 (13)
C12	0.0808 (13)	0.0764 (14)	0.1031 (17)	−0.0141 (11)	0.0639 (13)	−0.0013 (12)
C13	0.0835 (13)	0.0814 (14)	0.0652 (12)	−0.0113 (10)	0.0464 (10)	0.0005 (10)
C14	0.0579 (10)	0.0695 (11)	0.0538 (10)	−0.0107 (8)	0.0283 (8)	−0.0039 (8)

### Geometric parameters (Å, °)

**Table:**

O1—C6	1.3506 (18)	C10—C11	1.385 (3)
O2—C7	1.2456 (17)	C11—C12	1.355 (3)
O1—HO1	0.98 (3)	C12—C13	1.360 (4)
N1—C7	1.3311 (19)	C13—C14	1.381 (3)
N1—C8	1.466 (2)	C2—H2	0.956 (19)
N1—HN1	0.882 (18)	C3—H3	0.954 (19)
C1—C7	1.483 (2)	C4—H4	0.94 (2)
C1—C6	1.401 (2)	C5—H5	0.99 (2)
C1—C2	1.387 (2)	C8—H8A	1.010 (16)
C2—C3	1.375 (3)	C8—H8B	0.995 (17)
C3—C4	1.379 (3)	C10—H10	0.94 (2)
C4—C5	1.361 (3)	C11—H11	0.95 (3)
C5—C6	1.389 (3)	C12—H12	0.97 (3)
C8—C9	1.511 (3)	C13—H13	0.99 (2)
C9—C14	1.370 (2)	C14—H14	0.94 (2)
C9—C10	1.375 (3)		
			
O1···O2	2.4886 (19)	C9···H4^iv^	3.034 (17)
O1···N1^i^	2.9191 (18)	C14···H4^iv^	2.989 (17)
O2···C14	3.260 (3)	HN1···C2	2.554 (19)
O2···O1	2.4886 (19)	HN1···H2	2.00 (3)
O1···H2^i^	2.58 (2)	HN1···O1^iii^	2.083 (18)
O1···HN1^i^	2.083 (18)	H2···N1	2.597 (18)
O2···HO1	1.56 (3)	H2···HN1	2.00 (3)
O2···H8A	2.416 (18)	H2···O1^iii^	2.58 (2)
O2···H14	2.59 (2)	HO1···O2	1.56 (3)
O2···H5^ii^	2.692 (19)	HO1···C7	2.16 (2)
N1···O1^iii^	2.9191 (18)	HO1···H5^ii^	2.50 (3)
N1···H2	2.597 (18)	H4···C9^iv^	3.034 (17)
C1···C3^iv^	3.554 (2)	H4···C14^iv^	2.989 (17)
C1···C8^v^	3.549 (2)	H5···O2^vi^	2.692 (19)
C3···C1^iv^	3.554 (2)	H5···HO1^vi^	2.50 (3)
C6···C8^v^	3.507 (2)	H8A···O2	2.416 (18)
C7···C7^v^	3.400 (2)	H8A···H14	2.56 (2)
C7···C14	3.483 (3)	H8A···H13^vii^	2.54 (3)
C8···C1^v^	3.549 (2)	H8A···C1^v^	3.075 (18)
C8···C6^v^	3.507 (2)	H8A···C2^v^	3.060 (18)
C14···C7	3.483 (3)	H8B···H10	2.32 (3)
C14···O2	3.260 (3)	H8B···C6^v^	3.073 (17)
C1···H8A^v^	3.075 (18)	H10···H8B	2.32 (3)
C2···H8A^v^	3.060 (18)	H13···H8A^viii^	2.54 (3)
C2···HN1	2.554 (19)	H14···O2	2.59 (2)
C6···H8B^v^	3.073 (17)	H14···C7	3.09 (2)
C7···H14	3.09 (2)	H14···H8A	2.56 (2)
C7···HO1	2.16 (2)		
			
C6—O1—HO1	102.3 (13)	C9—C14—C13	121.17 (19)
C7—N1—C8	122.74 (13)	C1—C2—H2	118.7 (11)
C7—N1—HN1	119.3 (12)	C3—C2—H2	119.8 (11)
C8—N1—HN1	117.7 (13)	C2—C3—H3	118.8 (12)
C6—C1—C7	118.17 (12)	C4—C3—H3	121.6 (12)
C2—C1—C6	117.83 (15)	C3—C4—H4	120.2 (11)
C2—C1—C7	124.00 (14)	C5—C4—H4	119.2 (11)
C1—C2—C3	121.50 (16)	C4—C5—H5	122.9 (12)
C2—C3—C4	119.62 (17)	C6—C5—H5	116.9 (12)
C3—C4—C5	120.55 (19)	N1—C8—H8A	106.4 (11)
C4—C5—C6	120.15 (17)	N1—C8—H8B	105.6 (11)
C1—C6—C5	120.35 (14)	C9—C8—H8A	109.4 (10)
O1—C6—C1	121.46 (15)	C9—C8—H8B	108.8 (11)
O1—C6—C5	118.19 (15)	H8A—C8—H8B	114.1 (13)
O2—C7—C1	120.79 (13)	C9—C10—H10	117.9 (14)
O2—C7—N1	120.61 (14)	C11—C10—H10	121.6 (14)
N1—C7—C1	118.60 (12)	C10—C11—H11	118.5 (15)
N1—C8—C9	112.64 (13)	C12—C11—H11	121.0 (15)
C8—C9—C10	120.97 (16)	C11—C12—H12	123.9 (13)
C8—C9—C14	120.74 (16)	C13—C12—H12	116.2 (13)
C10—C9—C14	118.28 (18)	C12—C13—H13	122.5 (15)
C9—C10—C11	120.3 (2)	C14—C13—H13	117.6 (15)
C10—C11—C12	120.5 (2)	C9—C14—H14	120.7 (11)
C11—C12—C13	119.9 (2)	C13—C14—H14	118.2 (11)
C12—C13—C14	119.8 (2)		
			
C8—N1—C7—O2	1.6 (2)	C2—C3—C4—C5	−0.2 (3)
C8—N1—C7—C1	−178.95 (14)	C3—C4—C5—C6	0.2 (3)
C7—N1—C8—C9	89.06 (18)	C4—C5—C6—O1	179.22 (16)
C6—C1—C2—C3	0.4 (2)	C4—C5—C6—C1	0.1 (2)
C7—C1—C2—C3	−179.59 (16)	N1—C8—C9—C10	104.4 (2)
C2—C1—C6—O1	−179.48 (15)	N1—C8—C9—C14	−74.4 (2)
C2—C1—C6—C5	−0.4 (2)	C8—C9—C10—C11	−179.0 (2)
C7—C1—C6—O1	0.5 (2)	C14—C9—C10—C11	−0.2 (3)
C7—C1—C6—C5	179.56 (15)	C8—C9—C14—C13	178.77 (18)
C2—C1—C7—O2	175.27 (15)	C10—C9—C14—C13	−0.1 (3)
C2—C1—C7—N1	−4.2 (2)	C9—C10—C11—C12	0.1 (4)
C6—C1—C7—O2	−4.7 (2)	C10—C11—C12—C13	0.3 (4)
C6—C1—C7—N1	175.80 (14)	C11—C12—C13—C14	−0.6 (4)
C1—C2—C3—C4	−0.1 (3)	C12—C13—C14—C9	0.5 (3)

Symmetry codes: (i) *x*−1/2, −*y*+3/2, *z*−1/2; (ii) −*x*+1/2, *y*−1/2, −*z*−1/2; (iii) *x*+1/2, −*y*+3/2, *z*+1/2; (iv) −*x*+1, −*y*+2, −*z*; (v) −*x*+1, −*y*+1, −*z*; (vi) −*x*+1/2, *y*+1/2, −*z*−1/2; (vii) −*x*+3/2, *y*−1/2, −*z*−1/2; (viii) −*x*+3/2, *y*+1/2, −*z*−1/2.

### Hydrogen-bond geometry (Å, °)

**Table:**

*D*—H···*A*	*D*—H	H···*A*	*D*···*A*	*D*—H···*A*
N1—HN1···O1^iii^	0.882 (18)	2.083 (18)	2.9191 (18)	158.1 (18)
O1—HO1···O2	0.98 (3)	1.56 (3)	2.4886 (19)	157 (2)
C2—H2···O1^iii^	0.956 (19)	2.58 (2)	3.507 (2)	162.5 (15)
C14—H14···O2	0.94 (2)	2.59 (2)	3.260 (3)	128.8 (14)

Symmetry codes: (iii) *x*+1/2, −*y*+3/2, *z*+1/2.

## References

[bb1] Agwade, V. C. (1982). *Chem. Eng. Data*, **27**, 479–481.

[bb2] Allen, F. H., Kennard, O., Watson, D. G., Brammer, L., Orpen, A. G. & Taylor, R. (1987). *J. Chem. Soc. Perkin Trans. 2*, pp. S1–19.

[bb3] Higashi, T. (1995). *ABSCOR* Rigaku Corporation, Tokyo, Japan.

[bb4] Rigaku (1998). *RAPID-AUTO* Rigaku Corporation, Tokyo, Japan.

[bb5] Rigaku/MSC (2002). *CrystalStructure* Rigaku Corporation, Tokyo, Japan.

[bb6] Sheldrick, G. M. (2008). *Acta Cryst.* A**64**, 112–122. 10.1107/S0108767307043930 18156677

